# Suicidal behaviours among deaf adolescents in Ghana: a cross-sectional study

**DOI:** 10.1093/pubmed/fdab076

**Published:** 2021-04-06

**Authors:** E N B Quarshie, D Fobi, E K Acheampong, C M Honu-Mensah, J Fobi, O Appau, J Andoh-Arthur, K Oppong Asante

**Affiliations:** School of Psychology, University of Leeds, Woodhouse, LS2 9JT, Leeds, UK; Department of Psychology, University of Ghana, P.O. Box LG 84, Legon, Accra, Ghana; School of Education, University of Leeds, Woodhouse, LS2 9JT, Leeds, UK; Department of Special Education, University of Education, P.O Box 25, Winneba, Ghana; Department of Special Education, University of Education, P.O Box 25, Winneba, Ghana; Department of Special Education, University of Education, P.O Box 25, Winneba, Ghana; Department of Special Education, University of Education, P.O Box 25, Winneba, Ghana; Department of Psychology, University of Ghana, P.O. Box LG 84, Legon, Accra, Ghana; Department of Psychology, University of Ghana, P.O. Box LG 84, Legon, Accra, Ghana

**Keywords:** deaf adolescents, Ghana, suicide, suicidal attempt, suicidal ideation

## Abstract

**Background:**

A growing global concern is that suicide research has paid little attention to young people with disabilities, particularly, in low- and middle-income countries (LAMICs). We aimed to estimate the 12-month prevalence of suicidal ideation and attempt and describe some associations among deaf adolescents in Ghana.

**Methods:**

This is a cross-sectional anonymous self-report survey involving a nationally representative random sample of 450 school-going deaf adolescents. Data analysis included bivariate and multivariable approaches.

**Results:**

The overall 12-month prevalence of suicidal ideation was 19·3% (95% confidence interval [CI] = 15·8–23·3) and suicidal attempt was 15·6% (95% CI = 12·3–19·2). Although alcohol use and parental divorce were strongly associated with increased odds of both suicidal ideation and attempt, high subjective mental well-being was associated with reduced odds of both suicidal ideation and attempt. Living with no parents and being a final year student were associated with suicidal ideation, while male gender was associated with suicidal attempt.

**Conclusions:**

The prevalence of suicidal behaviours among school-going deaf adolescents in this study compares with estimates among in-school non-deaf adolescents in Ghana and other LAMICs in Africa, and also highlights the need for prevention efforts against the onset of suicidal ideation and possible transition to attempt and suicide among deaf adolescents.

## Introduction

The World Health Organization (WHO) defines suicidal behaviour as ‘a range of behaviours that include thinking about suicide (or ideation), planning for suicide, attempting suicide and suicide itself’.^1^ Globally, suicide is the second leading cause of death among 15–19-year-olds,^1^^,^^2^ with 79% of the annual world-wide suicide-related mortality reported in low- and middle-income countries (LAMICs).^1^^,^^3^^,^^4^ Across the general population, suicidal ideation is a strong risk for attempting suicide; attempted suicide represents the single significant risk for suicide.^1^^,^^5^^,^^6^

Given that disabilities are associated with negative physical and mental health outcomes, persons with functional disabilities—related to hearing and sight—have been found to be at an increased risk of attempted suicide, suicide and other emotional and behavioural problems.^7–12^ However, there is a growing global concern that suicide research has paid little attention to young people with disabilities, especially, in LAMICs: in particular, much of our understanding of suicidal behaviours among deaf adolescents is based on evidence from high-income countries.^9^^,^^11^^,^^13–18^

We performed and updated a systematic search for literature (We searched Global Index Medicus, Index Medicus for South-East Asia Region, PsycINFO, African Journals OnLine, MEDLINE and African Index Medicus up to July 2020, using keywords [e.g. (‘suicide ideation’ OR ‘suicidal ideation’ OR ‘self-harm ideation’ OR suicid* OR self-injur* OR mental) AND (deaf children OR ‘deaf adolescents’ OR ‘deaf young people’ OR disability OR adolescen* OR child* OR students OR teen* OR ‘young adults’ OR youth OR pupils)]. We did not apply any date or language restrictions. Our geographic search filter to identify countries in within low- and middle-income context included names of the countries in both English and languages relevant to the countries. We supplemented the searches by reviewing references and forward citations of relevant articles.) from Africa to contextualize the current study. Apart from relevant studies involving school-going non-deaf adolescents, we found neither published primary studies nor systematic reviews or meta-analyses with included studies involving deaf adolescents from African countries.^19–25^

Among in-school non-deaf adolescents, recent regional systematic reviews and meta-analyses suggest that relative to other LAMICs, the 12-month prevalence estimate of suicidal ideation (20.4%, 95% confidence interval [CI] = 17.3, 23.6) is higher in sub-Saharan Africa, while the pooled estimate of suicidal attempt (19.3%, 95%CI = 14.2, 24.4) is comparable to what pertains in LAMICs within the Western Pacific region (20.5%, 95%CI = 14.3, 26.7).^20–23^ The evidence also suggests that suicidal behaviours among adolescents are multicausal, with specific risk and protective factors existing at the personal level (e.g. female gender, alcohol and substance use, depression), family context (e.g. parental understanding, conflict with parents), school (e.g. schoolwork problems, bullying victimization) and other social relationship contextual factors (e.g. break-up, sexual abuse victimization, peer support).^21–23^

In Ghana, only one study has reported social discrimination as a critical reason for suicidal ideation among adult mothers with physical disabilities.^26^ Evidence also suggests that although deaf adolescents have access to health facilities in Ghana, professional health care is hampered by communication barriers such as the lack of sign language interpreters.^27^

Taken together, considering that the reduction of suicide-related deaths has been given special priority in the sustainable development goals, it is important to contribute sound and timely research evidence, particularly, among young vulnerable groups such as deaf adolescents in LAMICs to inform intervention and prevention efforts.

### Aims of study

(i) Estimate the 12-month prevalence of suicidal behaviours (Suicidal behaviours denote two outcomes in this study: suicidal ideation and suicidal attempt.) among school-going deaf adolescents (Schools for the deaf in Ghana are typically attended by young people with moderate to profound hearing loss. Based on the definition by Sawyer et al. (2018), we used the term adolescents to mean individuals aged 10–24 years. [Sawyer, S. M., Azzopardi, P. S., Wickremarathne, D., & Patton, G. C. (2018). The age of adolescence. *The Lancet Child & Adolescent Health, 2*(3), 223–228. doi:10.1016/S2352-4642(18)30022-1].) in Ghana.
(ii) Identify the common and unique factors associated with suicidal behaviours among school-going deaf adolescents in Ghana.

## Methods

### Design, setting and participants

**Fig. 1:**
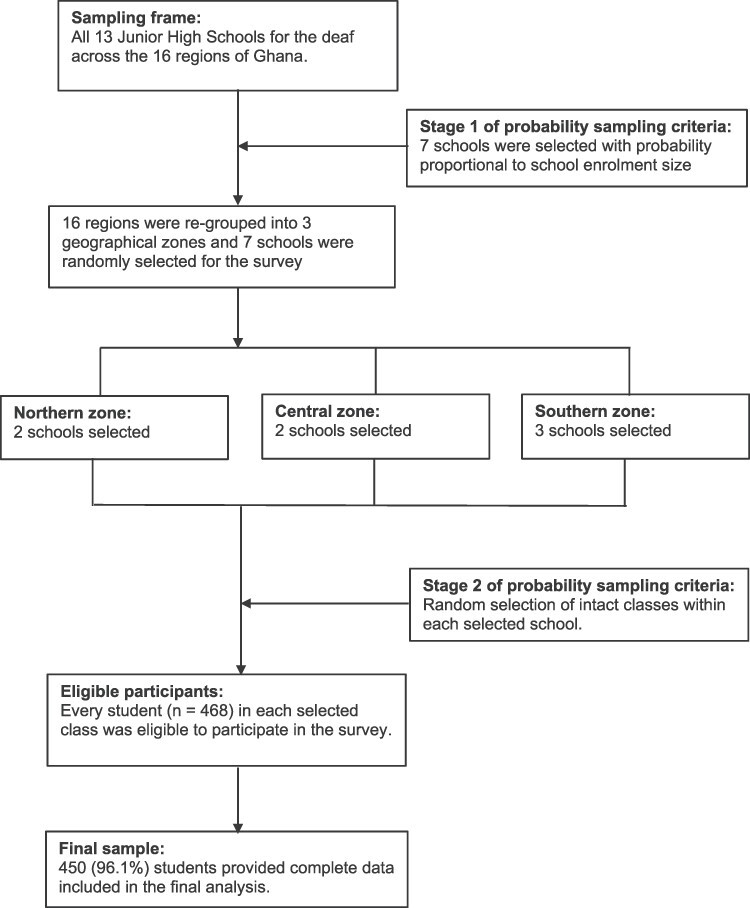
Summary of school selection and participant sampling for survey.

The community-agreed recommendations of Strengthening the Reporting of Observational Studies in Epidemiology (STROBE) guided the design and reporting of this study.^28^ We conducted a cross-sectional survey, using a self-report anonymous questionnaire, among deaf students attending Junior High Schools (JHSs) (In Ghana, Junior High Schools have a 3-year duration and are targeted at young people aged 12–14 years, but due to significant delayed school enrolment of young people with disabilities, typically, late and older adolescents aged 15–24 years are also predominantly found at this level of basic education in special schools in Ghana.) in Ghana. At the time of designing this study (August 2019) there were 1030 students across all the 13 JHSs for the deaf in the country. We predetermined a minimum sample size of 288, using Yamane’s (1967) formula for proportions, with 0·05 level of precision.^29^ We used a two-stage cluster sampling approach to firstly select seven JHSs for the deaf with probability proportional to enrolment size across the 16 administrative regions of Ghana. In stage 2, classes were randomly selected with all students in each class eligible to participate in the survey. Figure 1 summarizes the school selection and sampling of participants for this study. We approached and invited 468 participants, but 450 provided complete data included in the final analysis, representing a response rate of 96.1% (171 females and 221 males; aged 13–24 years; mean = 18.4; standard deviation = 2.60; modal age = 18).

### Outcome measures

Two outcome variables were included in this study: suicidal ideation and attempt. In order to facilitate comparison, we adopted two single-items from the 2012 Ghana WHO-Global School-based Health Survey (WHO-GSHS),^30^ one each to measure suicidal ideation and attempt. Specifically, the item, ‘during the past 12 months, did you ever seriously consider attempting suicide?’ was used to measure suicidal ideation; the responses were dichotomized as No (0) or Yes (1). Suicidal attempt was measured with the question ‘during the past 12 months, how many times did you actually attempt suicide?’ The responses were ‘0’, ‘1’, ‘2 or 3’, ‘4 or 5’ and ‘6 or more times’. Consistent with most published analyses of the WHO-GSHS data from sub-Saharan Africa,^20–24^ we applied a binary recoding to this item for analysis, No (no attempt = 0) and Yes (one or more attempts = 1).

### Exposure variables

This study involved 12 items assessing personal, family, school and interpersonal factors found in the 2012 Ghana WHO-GSHS to be associated with suicidal behaviours as risk and protective factors (see in Supplementary Table 1). For example, alcohol use (never drink alcohol or ≥1 drinks), parental divorce (no/yes), schoolwork problems (no/yes), bullying victimization (no/yes) and break-up (no/yes). Also, this study adopted one item from the 5-item Duke University Religion Index (DUREL) to measure *religious participation*: ‘How often do you attend church or other religious meetings?’ with response rating options ranging from (1) ‘never’ to (6) ‘more than once per week’.^31^ Considering the strong association between suicidal behaviours, and psychological functioning, life satisfaction and one’s ability to develop and maintain mutually beneficial social relationships,^32^ we adopted the 14-item Warwick-Edinburgh Mental Well-being Scale (WEMWBS) to assess subjective mental well-being.^13^^,^^33^ It is a 5-score scale, from ‘none of the time’ [1] to ‘all of the time’ [5] (e.g. ‘I’ve been feeling optimistic about the future’), with the possible total score ranging from 14 to 70 points. Higher scores indicate better mental well-being. Internationally, the WEMWBS has demonstrated strong internal consistency and a satisfactory Cronbach’s alpha;^34^ the Cronbach’s alpha in the current study was 0·83. Also, as shown in Supplementary Table 1, eight socio-demographic variables were included (e.g. gender, age, family structure). Prior to administration, the questionnaire was expert reviewed by two basic school Ghanaian Sign Language teachers and an adolescent suicide researcher.

### Procedure

The survey took place from October 2019 to January 2020. The survey was administered to participants in their school’s assembly hall or a larger classroom, with sitting arrangement spaced by reasonable distance to ensure privacy. We clarified participants’ queries in the Ghanaian Sign Language—the sign language that is used for the instruction and examination of deaf students at all levels of education in Ghana. Averagely, the completion of the questionnaire lasted between 55 and 75 min. After completing the survey, each student placed their answered questionnaire in an opaque box before exiting the hall.

### Data analysis

All data analyses were performed using the Statistical Package for Social Sciences (SPSS version 26.0 for Windows). As the loss of cases due to missing data was <5%, the list-wise deletion of missing data strategy was applied.^35^ The coding of the variables included in our statistical analysis and proportion of missing data are shown in Supplementary Table 1. The data analysis proceeded in two stages. Firstly, we performed descriptive analysis by applying frequencies, proportions and the Pearson’s Chi-square tests ($\chi^2\Big)$and point-biserial correlation (*r*_pb_) tests to examine the bivariate relationships between suicidal behaviours and the exposure variables and socio-demographic factors. Secondly, adjusted multivariable logistic regression analyses examining the possible associations between suicidal behaviours and the specified correlates and socio-demographic variables were performed. As recommended by leading logistic regression modelling experts, the candidate correlates were entered in the multivariable logistic regression models regardless of their statistically significant bivariate relationship with the outcome variables.^36–38^ The socio-demographic variables were included as covariates. We reported the results of the logistic regression as odds ratios with 95% confidence intervals (CI) and *P*-values. Statistically significant results were also determined using *P* < 0·05.

### Ethics

This study was approved by the Department of Psychology Research Ethics Committee, University of Ghana, Accra. We followed the ethical procedures of the Ghana Education Service (GES) for conducting research involving students in special schools in Ghana. The Special Education Unit of GES and heads of all the participating schools permitted this study. Each participant signed a written consent form prior to responding to the survey; parents/guardians of underage participants provided consent and we obtained the assent of participants younger than 18 years, prior to responding to the survey.

## Results

### Prevalence estimates and bivariate associations of suicidal behaviours

The overall 12-month prevalence estimate of suicidal ideation was 19·3% (95% CI = 15·8–23·3) and suicidal attempt was 15·6% (95% CI = 12·3–19·2). More males than females reported suicidal ideation (female = 17·0% [11·7–23·4]; male = 20·8% [95% CI = 16·2–26·0]) and attempt (female =12·3% [95% CI = 7·8–18·2]; male = 17·6% [95% CI = 13·3–22·5]). Overall, we found statistically significant bivariate associations between suicidal behaviours and most of the socio-demographic factors and exposure variables included in this study—see Tables 1 and 2.

**Table 1:** Chi-square tests assessing the bivariate associations, stratified by suicidal behaviour

		*Suicidal ideation in the previous 12 months*				*Suicidal attempt in the previous 12 months*			
*Variable*	*Sample distribution*	*No*	*Yes*			*No*	*Yes*		
	n (%)	n (%)	n (%)	$\chi 2$	*P*-value	n (%)	n (%)	$\chi 2$	*P*-value
Socio-demographic variables:									
Gender				0·997	·318			2·252	·133
Female	171 (38·0)	142 (83·0)	29 (17·0)			150 (87·7)	21 (12·3)		
Male	279 (62·0)	221 (79·2)	58 (20·8)			230 (82·4)	49 (17·6)		
Deafness status				8·083	·004			5·685	·017
Postnatal	168 (37·3)	124 (73·8)	44 (26·2)			133 (79·2)	35 (20·8)		
Congenital	282 (62·7)	239 (84·8)	43 (15·2)			247 (87·6)	35 (12·4)		
Grade:				3·239	·198			·691	·708
JHS 1	216 (48·0)	181 (83·8)	35 (16·2)			181 (83·8)	35 (16·2)		
JHS 2	73 (16·2)	59 (80·8)	14 (19·2)			64 (87·7)	9 (12·3)		
JHS 3	161 (35·8)	123 (76·4)	38 (23·6)			135 (83·9)	26 (16·1)		
Family structure				·002	·965				
Father has 1 wife	318 (72·6)	255 (80·2)	63 (19·8)			273 (85·8)	45 (14·2)	2·246	·134
Father has >1 wife	120 (27·4)	96 (80·0)	24 (20·0)			96 (80·0)	24 (20·0)		
Living arrangement:				12·592	·002			15·037	·001
Live with both parents	240 (54·4)	201 (83·8)	39 (16·3)			215 (89·6)	25 (10·4)		
Live with one parent	124 (28·1)	104 (83·9)	20 (16·1)			103 (83·1)	21 (16·9)		
Live with no parents	77 (17·5)	51 (66·2)	26 (33·8)			55 (71·4)	22 (28·6)		
In romantic relationship				4·643	·031			13·382	<·001
No	250 (56·3)	211 (84·4)	39 (15·6)			225 (90·0)	25 (10·0)		
Yes	194 (43·7)	148 (76·3)	46 (23·7)			150 (77·3)	44 (22·7)		
Religious group				7·232	·007			18·712	<·001
Christian	333 (74·5)	278 (83·5)	55 (16·5)			296 (88·9)	37 (11·1)		
Muslim	114 (25·5)	82 (71·9)	32 (28·1)			82 (71·9)	32 (28·1)		
Personal and lifestyle variables:									
Weekly alcohol use				29·037	<·001	302 (88·6)	39 (11·4)	21·204	<·001
Never drink alcohol	341 (77·0)	293 (85·9)	48 (14·1)			71 (69·6)	31 (30·4)		
≥1 drink	102 (23.0)	63 (61·8)	39 (38·2)						
Family factors:									
Parental divorce				24·652	<·001			20·079	<·001
No	296 (66·8)	258 (87·2)	38 (12·8)			266 (89·9)	30 (10·1)		
Yes	147 (33·2)	99 (67·3)	48 (32·7)			108 (73·5)	39 (26·5)		
Conflict with parents				7·907	·005			10·023	·002
No	277 (62·7)	235 (84·8)	42 (15·2)			246 (88·8)	31 (11·2)		
Yes	165 (37·3)	122 (73·9)	43 (26·1)			128 (77·6)	37 (22·4)		
School factors:									
Schoolwork problems				4·813	·028			4·184	·041
No	129 (29·1)	112 (86·8)	17 (13·2)			116 (89·9)	13 (10·1)		
Yes	314 (70·9)	244 (77·7)	70 (22·3)			258 (82·2)	56 (17·8)		
Bullying victimization				8·368	·004			6·071	·014
No	202 (44·9)	175 (86·6)	27 (13·4)			180 (89·1)	22 (10·9)		
Yes	248 (55·1)	188 (75·8)	60 (24·2)			200 (80·6)	48 (19·4)		
Interpersonal adversities:									
Break-up	298 (66·2)			2·786	·095			5·280	·022
No	152 (33·8)	247 (82·9)	51 (17·1)			260 (87·2)	38 (12·8)		
Yes		116 (76·3)	36 (23·7)			120 (78·9)	32 (21·1)		
Physical abuse victimization				37·398	<·001			15·445	<·001
No	292 (64·9)	260 (89·0)	32 (11·0)			261 (89·4)	31 (10·6)		
Yes	158 (35·1)	103 (65·2)	55 (34·8)			119 (75·3)	39 (24·7)		
Sexual abuse victimization				28·224	<·001			25·863	<·001
No	365 (82·6)	310 (84·9)	55 (15·1)			322 (88·2)	43 (11·8)		
Yes	158 (35·1)	45 (58·4)	32 (41·6)			50 (64·9)	27 (35·1)		

Note: $\chi 2$ = Chi square

**Table 2:** Point-biserial tests assessing the bivariate associations, stratified by suicidal behaviour

	*Suicidal ideation in the previous 12 months*			*Suicidal attempt in the previous 12 months*		
*Variable*	*n*	*r_pb_*	**P*-value*	*n*	*r_pb_*	**P*-value*
Socio-demographic variables:						
Age	450	·076	·106	450	·095	·044
Personal and lifestyle variables:						
Subjective mental well-being	450	−·250	<·001	450	−·282	<·001
Religious participation	444	−·132	·005	444	−·163	·001
Family factors:						
Parental checking of homework	443	−·154	·001	443	−·064	·177
Parental understanding	447	−·125	·008	447	−·123	·009
Parental monitoring	445	−·063	·187	445	−·014	·775
Parental intrusion of privacy	446	−·018	·712	446	−·037	·434

Note: r_pb_ = point biserial coefficient

Deaf adolescents who reported physical abuse victimization, weekly alcohol use, sexual abuse victimization and parental divorce were more likely to report suicidal ideation and attempt during the previous 12 months (Table 1). However, participants who reported high subjective mental well-being, frequent religious participation and high parental understanding were less likely to report both suicidal ideation and attempt (Table 2).

### Multivariable associations

As shown in Table 3, although weekly alcohol use and parental divorce were strongly associated with increased odds of both suicidal ideation and attempt, high subjective mental well-being emerged as a strong correlate of reduced odds of both suicidal ideation and attempt. While living with no parents (AOR = 2·358; 95% CI = 1·027, 5·414, *P* = ·043), being in JHS 3 (AOR = 2·145; 95% CI = 1·004, 4·584, *P* = ·049), and being deaf postnatally (AOR = 0·408; 95% CI = 0·205, 0·811, *P* = ·011) were uniquely associated with increased odds of suicidal ideation, frequent parental checking of homework (AOR = 0·601; 95% CI = 0·448, 0·806, *P* = ·001) was associated with reduced odds of suicidal ideation. Males were about two times likely than females to report suicidal attempt during the previous 12 months (AOR = 2·262; 95% CI = 1·032, 4·961, *P* = ·042)—see Table 3.

**Table 3:** Multivariate associations, stratified by suicidal behaviour

	*Suicidal ideation in the previous 12 months*				*Suicidal attempt in the previous 12 months*			
*Variables in model*	*β*	*AOR*	*95% CI*	**P*-value*	*β*	*AOR*	*95% CI*	**P*-value*
Socio-demographic variables:								
Gender	·434	1·543	·767, 3·105	·224	·816	2·262	1·032, 4·961	·042
Age	·000	1·000	·866, 1·155	·998	·038	1·038	·888, 1·215	·638
Deafness status	−·897	·408	·205, ·811	·011	−·386	·680	·322, 1·437	·312
Grade:								
JHS 1	Reference				Reference			
JHS 2	·870	2·387	·947, 6·014	·065	·202	1·224	·433, 3·462	·704
JHS 3	·763	2·145	1·004, 4·584	·049	−·044	·957	·420, 2·184	·917
Family structure	−·458	·632	·296, 1·349	·236	·129	1·138	·522, 2·479	·745
Living arrangement:								
Live with both parents	Reference				Reference			
Live with one parent	−·721	·486	·216, 1·095	·082	·305	1·357	·602, 3·058	·462
Live with no parents	·858	2·358	1·027, 5·414	·043	·891	2·437	·971, 6·116	·058
In romantic relationship	·057	1·059	·499, 2·249	·881	·100	1·105	·493, 2·480	·808
Religious group	−·200	·819	·383, 1·749	·606	·608	1·836	·846, 3·986	·125
Personal and lifestyle variables:								
Subjective mental well-being	−·052	·949	·915, ·984	·005	−·079	·924	·884, ·965	<·001
Religious participation	−·038	·963	·806, 1·150	·676	−·121	·886	·736, 1·067	·201
Weekly alcohol use	·950	2·585	1·211, 5·520	·014	1·010	2·745	1·251, 6·025	·012
Family factors:								
Parental divorce	·792	2·208	1·121, 4·346	·022	·779	2·180	1·028, 4·623	·042
Conflict with parents	·185	1·203	·594, 2·438	·607	·338	1·402	·652, 3·015	·386
Parental checking of homework	−·510	·601	·448, ·806	·001	−·123	·885	·648, 1·207	·439
Parental understanding	−·019	·981	·744, 1·294	·892	−·001	·999	·725, 1·376	·994
Parental monitoring	·026	1·026	·788, 1·336	·850	·132	1·142	·840, 1·552	·398
Parental intrusion of privacy	·106	1·112	·867, 1·425	·403	·005	1·005	·758, 1·331	·975
School factors:								
Schoolwork problems	·405	1·500	·674, 3·338	·321	·305	1·356	·564, 3·264	·496
Bullying victimization	·233	1·262	·626, 2·543	·515	·069	1·071	·496, 2·312	·861
Interpersonal adversities:								
Break-up	−·293	·746	·341, 1·630	·463	−·287	·750	·329, 1·713	·495
Physical abuse victimization	1·273	3·572	1·795, 7·108	·000	·430	1·537	·728, 3·244	·260
Sexual abuse victimization	1·029	2·798	1·253, 6·250	·012	·678	1·969	·832, 4·663	·123
Nagelkerke pseudo R^2^		·433				·402		
Cox & Snell R^2^		·272				·229		
Hosmer-Lemeshow GOF test (sig·)		3·93 (·863)				7·66 (·467)		
Overall percentage correctly classified		85·8				88·7		

Note: *β* = beta value; AOR = adjusted odds ratios; CI = Confidence Interval; GOF = goodness of fit

## Discussion

### Main findings of this study

Across the total sample, nearly two in 10 participants reported suicidal ideation, while one out of 10 participants reported suicidal attempt in the previous 12 months. The factors associated with suicidal ideation and attempt were multi-contextual: personal, family and school related. Although alcohol use and parental divorce were strongly associated with increased odds of both suicidal ideation and attempt, high subjective mental well-being was associated with reduced odds of both suicidal ideation and attempt. Living with no parents, being a final year student and being deaf postnatally were strongly associated with increased odds of suicidal ideation, while male gender was associated with increased odds of suicidal attempt. Although age showed positive associations with suicidal ideation and attempt, these associations did not reach the desired statistical significance.

### What is already known on this topic

Most of what is already known about this topic is from high-income context, with earlier systematic reviews identifying no studies from LAMICs.^8^^,^^13^ Earlier evidence from high-income countries suggests that lifetime estimates of suicidal ideation among deaf adolescents ranges widely between 4.6 and 44%, while 12-month attempted suicide ranges between 1.7 and 18%.^13^ Recently, a county-wide cross-sectional study from the USA has reported a 12-month prevalence estimates of attempted suicide (14.9%) among deaf adolescents.^11^ Generally, major depression, family dysfunction, low academic performance, alcohol use, peer victimization and peer relationship difficulties and sexual abuse victimization have been reported as significant associations of suicidal ideation and attempt among adolescents with functional disabilities in high-income countries.^11^^,^^13^^,^^14^^,^^39^ The only available study reporting evidence from a low-income country is a recent school-based cross-sectional survey from Pakistan that reports lifetime suicidal attempt (19%) and 12-month suicidal ideation (17%) among school-going deaf adolescents.^15^

### What this study adds

This study represents a pioneering attempt at providing evidence on suicidal behaviours among deaf adolescents from Ghana; the evidence could be potentially informative for more expansive epidemiological studies from Ghana and other sub-Saharan African countries. Although the prevalence estimates and associated factors of suicidal behaviours in the current study are comparable to those reported among in-school non-deaf adolescents in Ghana and other countries in sub-Saharan Africa,^19–24^ the adjusted multivariate logistic models of the current study have shown two new specific evidence: (1) there is a significant negative association between subjective mental well-being and both suicidal ideation and attempt, and (2) adolescents with congenital deafness are less likely to report suicidal ideation, compared to adolescents of postnatal deafness. These underscore the need for universal and targeted prevention efforts aimed at improving the mental well-being of school-going deaf adolescents in Ghana. Put together, although further studies (including qualitative research) are needed to explore the lived experiences and meanings of suicidal behaviours among school-going deaf adolescents in Ghana, the evidence of the current study could be pointing to the possibility that both adolescents without disabilities and those with disabilities may be faced with common multiple distress and challenges of health, education, growth and development, and family and social adversities in sub-Saharan Africa.^40–44^

### Limitations of this study

Considering that in cross-sectional surveys both the outcome and exposure variables are measured at the same point in time, it stands to suggests that the evidence reported in this study cannot support causal inferences.^45^ Although the use of single-item measures affords the opportunity of screening a larger sample at one point in time for suicidal behaviours, single-item measures have the potential of leading to misclassification of suicidal behaviour.^46^^,^^47^ Relatedly, the self-report approach used (instead of a diagnostic approach) might have yielded less reliable responses in respect of suicidal ideation and attempt. Furthermore, besides the high tendency of nondisclosure among young people in suicide research,^48^ some participants in this study might have also provided socially desirable responses due to the highly stigmatized and criminalized nature of attempted suicide in Ghana.^49^^,^^50^ While these limitations provide a critical basis for cautious interpretations of the findings, the inclusion of a nationally representative sample of deaf students in JHSs in Ghana could make the findings a good reflection of the national prevalence and associated factors of suicidal ideation and attempt among deaf students attending JHSs.

## Conclusions

The prevalence of suicidal ideation and attempt among deaf adolescents in this study compares with estimates among non-deaf adolescents in Ghana and other LAMICs in Africa, but also highlights the need for targeted and universal prevention efforts against the onset of suicidal ideation and possible transition to attempt and suicide among deaf adolescents.

## Data availability statement

The data underlying this article will be shared on reasonable request to the corresponding author.

## Conflict of interest

The authors declare that they have no competing interests.

## Funding

This research received no specific grant from any funding agency in the public, commercial or not-for-profit sectors.

## Authors’ contributions

E.Q. and D.F. contributed to the study concept and design; E.A., C.H.-M., J.F. and O.A. conducted the data collection; E.Q. performed statistical analysis of the data; J.A.-A. and K.O.A. contributed to the interpretation of the results; E.Q., D.F., E.A., C.H.-M., J.F. and O.A. drafted the manuscript and D.F., E.A., J.A.-A. and K.O.A. critiqued the manuscript for important intellectual content. All the authors contributed to the revision of the manuscript and approved the final version.

## Supplementary Material

Supplementary_material_fdab076

## Notes

**Emmanuel Nii-Boye Quarshie,** Research fellow in youth self-harm and suicide prevention

**Daniel Fobi,** PhD candidate in deaf education

**Emmanuel Kwasi Acheampong,** Lecturer in deaf education

**Cyril Mawuli Honu-Mensah,** Lecturer in audiology

**Joyce Fobi,** Teaching assistant in deaf education

**Obed Appau,** Principal research assistant in deaf education

**Johnny Andoh-Arthur,** Lecturer in community psychology and suicidology

**Kwaku Oppong Asante,** Senior lecturer in health psychology
