# Supplementary material for: Suicidal behaviours among deaf adolescents in Ghana: a cross-sectional study
Source: J Public Health (Oxf). 2021 Apr 6;44(1):e10–9. doi: 10.1093/pubmed/fdab076 (PMC8904192; doi:10.1093/pubmed/fdab076)
Supplement: Supplementary_material_fdab076 [file supplementary_material_fdab076.docx]

**Supplementary material**

eTable 1. Coding of variables and missing data

| **Variables** | **Survey question** | **Response coding** | **Observation / Missing (%)** |
| --- | --- | --- | --- |
| **Socio-demographic variables:** |  |  |  |
| Gender | What is your gender? | Female = 0; Male = 1 | N = 450 / 0 (0) |
| Age | What is your age? | Coded continuously (13 – 24 years) | N = 450 / 0 (0) |
| Deafness status | Where you born deaf or you used to hear? | Postnatal = 0  Congenital = 1 | N = 450 / 0 (0) |
| School grade | In which form are you? | JHS 1 = 0  JHS 2 = 1  JHS 3 = 2 | N = 450 / 0 (0) |
| Family Structure | How will you describe your family structure? | My father has 1 wife = 0  My father has > 1 wife = 1 | N = 438 / 12 (2.7) |
| Living arrangement | What is your living arrangement? I live… | with both parents = 0  with one parent = 1  with no parents = 2 | N = 441 / 9 (2.0) |
| In romantic relationship | Do you have a boyfriend or girlfriend? | No = 0; Yes = 1 | N = 444 / 6 (1.3) |
| Religious group | What is your religious group? | Christian = 0; Muslim = 1 | N = 447 / 3 (0.7) |
| **Personal & lifestyle variables:** |  |  |  |
| Subjective mental wellbeing | Assessed using the Warwick-Edinburgh Mental Well-being Scale | 14-item scale scored continuously:  None of the time (1) – All of the time (5) | N = 450 / 0 (0) |
| Religious participation | How often do you attend church or other religious meetings? | (coded continuously):  Never = 1  Once a year or less = 2  A few times a year = 3  A few times a month = 4  Once a week = 5  More than once/week = 6 | N = 444 / 6 (1.3) |
| Weekly alcohol use | How many alcoholic drinks do you have in a typical week? | Never drink = 0  1 or more drinks = 1 | N = 443 / 7 (1.6) |
| **Family factors:** |  |  |  |
| Parental divorce | Have your parents separated or divorced during the past 12 months? | No = 0; Yes = 1 | N = 443 / 7 (1.6) |
| Conflict with parents | Have you had any serious arguments or fights with either or both of your parents during the past 12 months? | No = 0; Yes = 1 | N = 442 / 8 (1.8) |
| Parental checking of homework | During the past 30 days, how often did your parents or guardians check to see if your homework was done? | 1 = never to 5 = always | N = 443 / 7 (1.6) |

eTable 1. (continued)

| Parental understanding | During the past 30 days, how often did your parents or guardians understand your problems and worries? | 1 = never to 5 = always | N = 447 / 3 (0.7) |
| --- | --- | --- | --- |
| Parental monitoring | During the past 30 days, how often did your parents or guardians really know what you were doing you’re your free time? | 1 = never to 5 = always | N = 445 / 5 (1.1) |
| Parental intrusion of privacy | During the past 30 days, how often did your parents or guardians go through your things without your approval? | 1 = never to 5 = always | N = 446 / 4 (0.9) |
| **School factors:** |  |  |  |
| School work problems | Have you had problems keeping up with schoolwork during the past 12 months? | No = 0; Yes = 1 | N = 443 / 7 (1.6) |
| Bullying victimisation | Have you been bullied at school or in your area during the past 12 months? (Bullying occurs when a young person or group of young persons tease, threaten, spread rumours about, hit, shove, or hurt another young person over and over again. It is not bullying when two young persons of about the same strength or power argue or fight or tease each other in a friendly and fun way)? | No = 0; Yes = 1 | N = 450 / 0 (0) |
| **Interpersonal adversities:** |  |  |  |
| Breakup | Have you had a break-up with a boyfriend or girlfriend during the past 12 months? | No = 0; Yes = 1 | N = 450 / 0 (0) |
| Physical abuse victimisation | Have you been seriously physically beaten during the past 12 months? | No = 0; Yes = 1 | N = 450 / 0 (0) |
| Sexual violence victimisation | Has anyone forced you (i.e. physically or verbally) to engage in sexual activities against your will during the past 12 months? | No = 0; Yes = 1 | N = 442 / 8 (1.8) |
| **Outcome variables:** |  |  |  |
| 12-month suicidal ideation | During the past 12 months, did you ever seriously consider attempting suicide? | No = 0; Yes = 1 | N = 450 / 0 (0) |
| 12-month suicide attempt | During the past 12 months, how many times did you actually attempt suicide? | Scored continuously, 0 – 6, but re-coded into:  0 = No  1 = Yes (1 or more times) | N = 450 / 0 (0) |
